# Open-source perfusion system for medium-scale fabrication of demineralized bone matrix chip grafts

**DOI:** 10.1016/j.ohx.2022.e00378

**Published:** 2022-11-23

**Authors:** Winston Jaramillo-Cañas, Frank Britto-Bisso, Cesar Fernandez-Valiente, Fanny L. Casado

**Affiliations:** a Tissue Bank, Hospital Luis Vernaza, Guayaquil, Ecuador; b Program of Biomedical Engineering PUCP-UPCH, Pontificia Universidad Catolica del Peru, Avenida Universitaria 1801, Lima 15088, Peru; c Institute of Omics Sciences and Applied Biotechnology, Pontificia Universidad Catolica del Peru, Avenida Universitaria 1801, Lima 15088, Peru

**Keywords:** Perfusion system, Bone grafting, Tissue Engineering

## Abstract

Demineralized bone matrix (DBM) is considered one of the most reliable bone tissue grafts for regular surgical use, as it provides a scaffold that is structurally like native bone, and that enhances bone regeneration. However, commercially available DBM products are not suited for surgical restitutions of large bones. Therefore, each Tissue Bank is urged to implement their own demineralization protocol, which usually does not meet the high demand for bone grafting. In this project, we developed an open source system for medium-scale manufacturing of DBM grafts from human cadaveric donors to automate the demineralization protocol and improve its reproducibility. The device consists in (1) unidirectional flow reaction chamber, where the demineralization protocol takes place; (2) automated syringe pump, which controls the reagent́s inlet and vacuum; and (3) reagent dispenser, for the management of the reagents need for the demineralization protocol. Validation of the device included histological analysis, DNA quantification temperature regulation, electrochemiluminescence and colorimetric protocols, followed by the optimization of physicochemical parameters based on Response Surface Methodology. The results showed values of residual lipids and calcium within standardized ranges, and the maintenance of the structural integrity of the DBM, demonstrating the capacity of the system to support the proposed demineralization protocol.

## Graphical abstract

**Figure:**
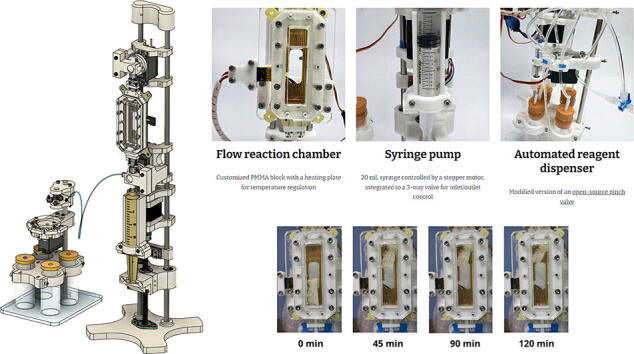

## Specifications table

**Table:** 

Hardware name	*Perfusion system for the fabrication of demineralized bone tissue chip grafts*
Subject area	● Engineering and materials science ● Medical (e.g., Tissue Engineering)
Hardware type	● Biological sample handling and preparation
Closest commercial analog	*“No commercial analog is available.”*
Open source license	BSD-2
Cost of hardware	∼ $340
Source file repository	*Uploaded at the time of submission on the online Elsevier submission interface (.zip)* *Zenodo data:**https://doi.org/10.5281/zenodo.6795588*

## Hardware in context

Surgical restitution of large bone lesions through orthopedic, cranio-maxillofacial and reconstructive procedures needs a biomaterial capable of fixing the scattered bone segments into their correct anatomical position while compensating the loss of bone mass. Bone grafting, the surgical procedure to transplant bone tissue, is currently used for repairing defects after fractures and tumor resections [1], [2] and in spinal fusion procedures [3], [4]. Currently, the main available bone grafts present some major drawbacks. Autologous grafts, when the donor and host are the same person, are limited by the harvesting methods (i.e. anatomical limitations and donor site morbidity), while allografts may present antigens from the donor that increase the risk of an adverse immune and inflammatory response [5]. Therefore, the ideal material for reconstruction of bone defects requires a biocompatible graft with low donor site morbidity, structural similarity to the native bone and that is readily accessible for surgeons. In this context, demineralized bone matrix (DBM) emerges as an allogenic alternative to autologous grafts that avoids the disadvantages of allografts due to its fabrication method.

DBM is a cell-free scaffold made up of proteins (mainly collagen), a variable percent of residual calcium phosphates, a small percent of cellular debris and biological drivers for bone regeneration such as growth factors and bone morphogenic proteins, which are known to be essential for osteogenesis [6], [19]. Basically, DBM is the protein matrix that remains after the decellularization of the adherent soft tissue, and the removal of lipids, blood, and mineral components from the raw trabecular bone. The purpose of this process is the removal of immunogenic cellular components, while preserving the structural, biochemical, and biomechanical integrity of the protein matrix [5]. DBM is considered one of the most reliable methods for regular clinical use, as it provides a scaffold that is structurally like native bone, and that enhances osteoconduction, osteoinduction and osteogenesis, thus allowing a complete integration to the adjacent host bone [7], [8].

These properties have allowed DBM to be extensively used for bone tissue engineering (BTE) applications that involves enhancing bone repair by considering the traditional BTE triad (scaffolds, cells and growth factors) for advanced defect bone reconstruction. DBM-based scaffolds have shown to promote osteoprogenitor cells adhesion, viability, proliferation and differentiation [8]. Moreover, since demineralization of trabecular bone yields a soft material, DBM-based scaffolds have been enhanced by cross-linking and hybrid scaffold fabrication, resulting in a biomaterial with the optimal mechanical strength, and pore size and distribution [7]. Ultimately, stem cells are seeded in these enhanced DBM-based scaffolds and maintained in bioreactors capable of providing dynamic culture conditions for homogeneous nutrient transport, gas exchange and metabolic waste discharge that allowed increased in cell viability, homogeneous distribution of cells within the scaffold, and proliferation. In particular, perfusion bioreactors for BTE provide also mechanical cues and native-like shear stress through the control of fluid flow to commit stem cells into a bone phenotype [9]–[12].

The manufacture of DBM can be summarized in the following steps: (1) gross cleaning and lipid removal, (2) demineralization, and (3) sterilization and preservation. Following these steps, the most common product of bone demineralization is DBW powder [9], which can be difficult to manage clinically. Therefore, different carriers have been used to facilitate handling of DBM composites. For instance, commercially available DBM products used hydrogels (e.g. Optecure ®), gelatin (e.g. Altiva®, Optefil®), hyaluronic acid (e.g. DBX®) and poloxamer reverse phase medium (e.g. Accell Connexus®, Allofuse®, DynaGraft II®, OrthoBlast®) as carriers [1], [6]. However, DBM content among these products are highly variable due to different preparation and processing methods, which might impact their clinical performance. Also, their presentations in gel, putty, paste, sheets, and strips are often not suitable for surgery of large bone lesions. Furthermore, Gruskin et al. suggested that individual DBM products prepared manually in the same tissue bank may present different osteoinductive potentials [6], exhibiting the need for the standardization of demineralization protocols.

To define the requirements for a typical medium-scale production environment of DBM, we systematized information provided by the Tissue Bank at the Luis Vernaza Hospital in Guayaquil, Ecuador. Thus, despite having an implemented demineralization protocol [13], DBM production is limited by the following aspects:

• **Availability of starting material versus demand.** Trabecular bone from cadaveric human donors is a high-demand product on most Tissue Banks and exhibits a complex harvesting procedure. The clinical demand for large pieces and powder presentations is mostly covered by large manufacturing facilities. However, there is a high demand for chips sized 12 × 24 × 5 mm that a medium-scale facility could attend but no optimized procedures.
• **Lack of specialized equipment.** State-of-the-art assays [14] are not reproducible due to the lack of equipment for handling the chips in particular vacuum, temperature, flow, and sterile conditions.
• **Density changes in bone tissue.** The ${\mathrm{CO}}_{2}$ generated during the processing of chips prevents a homogeneous demineralization in the trabeculae.
• **Lipid cross-contamination of products.** Mechanical agitation during the cleaning process generates lipid emulsifications that contaminate the chips.

Considering these requirements, the optimal design for a DBM fabrication device would be perfusion-based. Adjustable flow rate [9], [10], [15] allow the fine-tuning of reagentś flow and shear stress through each trabeculae that constitutes the microstructure of trabecular bone, avoiding emulsifications during the cleaning step and a homogeneous demineralization process. The most common perfusion-based systems for bone tissue engineering applications are bioreactors implemented to support cell culture seeded in a 3D scaffold. Peristaltic and syringe pumps are used to control flow rate (up to 5 mL/min) of culture medium and the removal of metabolic waste in a closed-loop platform fabricated using rapid manufacturing techniques [11], [15], [16]. This setup enables dynamic culture conditions that have shown to improve proliferation and viability of human bone marrow stromal cells [9], [10] and stem-cell derived mesenchymal progenitors [12]. Likewise, these systems can provide mechanical cues and native-like shear stress through the control of fluid flow and compressive loads that generate a favorable environment for osteogenic commitment [9], [15].

Nevertheless, we have identified an absence of scientific literature and commercially available devices focused on the design and fabrication of equipment for medium-scale DBM manufacture [20], here we describe the fabrication and performance of a perfusion system to implement a medium-scale manufacture protocol of DBM chips from human cadaveric donors that provides ideal experimental conditions for trabecular bone demineralization in terms of pulse washing, adjustable reaction temperature, and management of reagents. Thus, we address the mentioned challenges while improving the reproducibility of the demineralization protocol for medium-scale production conditions.

## Hardware description

**Fig. 1:**
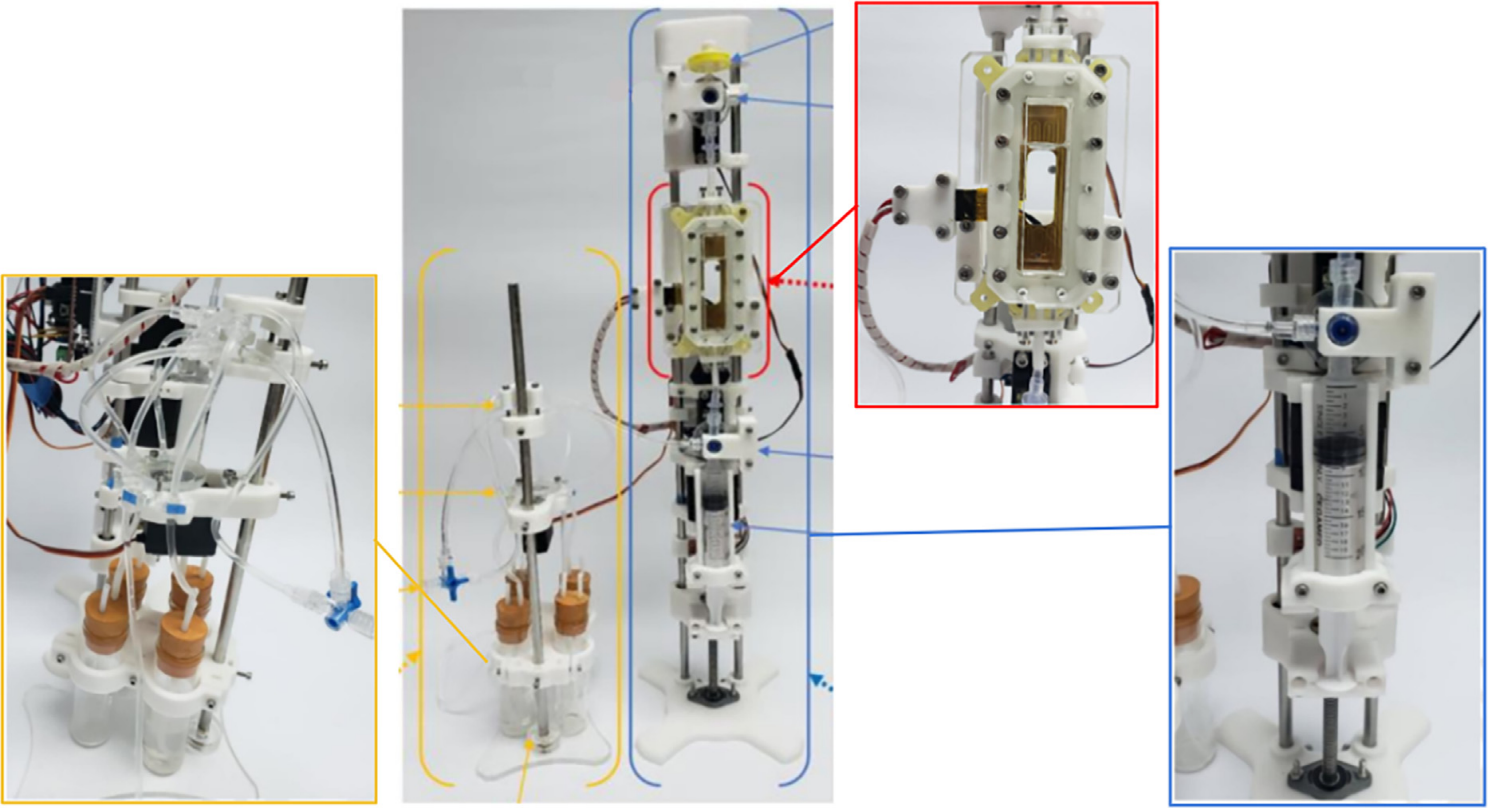
Overview of the main components of the perfusion system.

The system consists of three major components: a unidirectional flow reaction chamber, a syringe pump, and an automated reagent dispenser, all of them controlled simultaneously by the firmware (Fig. 1). The reaction chamber consists of an acrylic block customized using a CNC router, adhered to a heating plate for temperature control, with direct connections to the other components. The syringe pump comprises a 20 mL syringe controlled by a bipolar stepper motor integrated to a three-way stopcock at the tip of the syringe, controlled by a servomotor for reagent́s inlet and vacuum control. Finally, the reagent dispenser consists in a modified version of an open-source pinch valve [17], integrated with a servomotor for the control of five hoses connected to containers with the reactants needed for the demineralization process. Important features are mentioned in Table 1.

**Table 1:** Technical specifications of the system.

Parameters	Description
Energy	Universal power supply for 100–245 V AC/50–60 Hz, 560 W
Total weight	1.7 kg
Size	**Flow microreactor & Syringe pump:** 56 × 15 × 15 cm **Reaction chamber:** 12.5 × 5.5 × 2 cm (12 ${\mathrm{cm}}^{3}$ effective volumetric capacity) **Reagent dispenser:** 34 × 10 × 15 cm
Temperature	Heating plate temperature range: 25–100 °C, with a work temperature of 60 °C. Temperature is controlled by NTC thermistor (100 K) with a precision of ± 0.4 °C from 0 to 60 °C.
Pressure	Work pressure on the inside of the reaction chamber: − 240 mmHg
Number of ports	Up to 5 ports*
Mixture mechanism	Pulsating piston stirring at a frequency of five pulses per minute

(*) Number of ports can be changed by modifying the pinch valve design.

The design process of the system took into consideration the following requirements:

• Handling of tissue samples of size 12 × 24 × 5 mm
• Implementation of a pulse washing system that works unidirectionally
• Adequate visualization of the reaction chamber for image acquisition
• Temperature control within a range of 25 to 70 °C
• Resistance to acid and non-halogenated organic solvents; specifically, hydrochloric acid, hydrogen peroxide at 30 % and isopropyl alcohol
• Tolerate vacuum of −700 mmHg
• Reusability and easiness of cleaning to meet biosafety protocols of the Tissue Bank

Based on previous literature [18]–[21], the present project proposes and validates a washing procedure with a saline solution injected by pulses (five per minute), followed by a chemical treatment with hydrogen peroxide at 3 % and isopropyl alcohol at 100 % for degreasing and decellularization. Finally, based on the results of Pang et al (2021), we consider the acidic treatment with hydrochloric acid at 0.5 N for the demineralization step [22].

### Flow reaction chamber

**Fig. 2:**
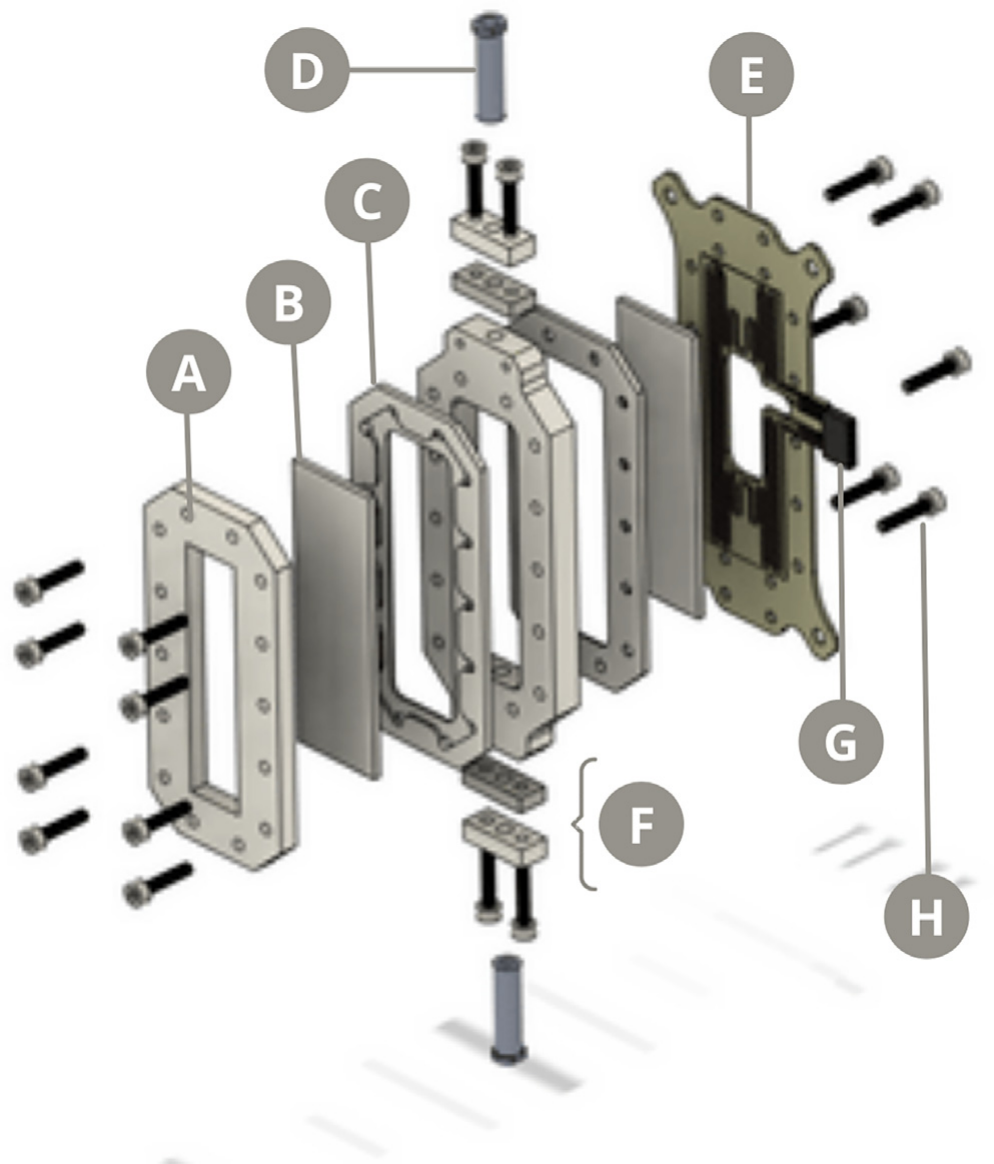
Exploded view diagram of the reaction chamber. A) Main lid B) Glass slide C) Silicone cover D) Luer lock E) Heating plate F) Adaptors G) NTC thermistor H) M3 screws.

The main component of the reaction chamber is a 44 × 108 × 8 mm piece of transparent PMMA customized using the CNC, reinforced with an anterior and posterior silicone cover, and with superior and inferior Luer locks for the connection to the vacuum valve and the syringe pump, respectively (Fig. 2). The assembly of these pieces leaves room for a chamber where the bone chips are placed, covered by an anterior and posterior microscope slide to fixate the sample to the chamber. An acrylic cover is fixated with M3 screws to the anterior part of this assembly. Likewise, the posterior part of the chamber is adhered to a PCB circuit with an epoxy heating plate with a customized design generated with the CNC. A NTC thermistor is placed in the PCB for temperature control.

The superior part of the reaction chamber is connected through the Luer lock to the vacuum valve. This component comprises a three-way stopcock controlled by a servomotor that connects the reaction chamber to a vacuum pump and an air exhaust for depressurization, following the same design as the flow valve in the syringe pump (see Section 2.2 Syringe pump). To maintain a sterile environment, an air filter is attached between the air inlet and the reaction chamber Luer lock.

### Syringe pump

**Fig. 3:**
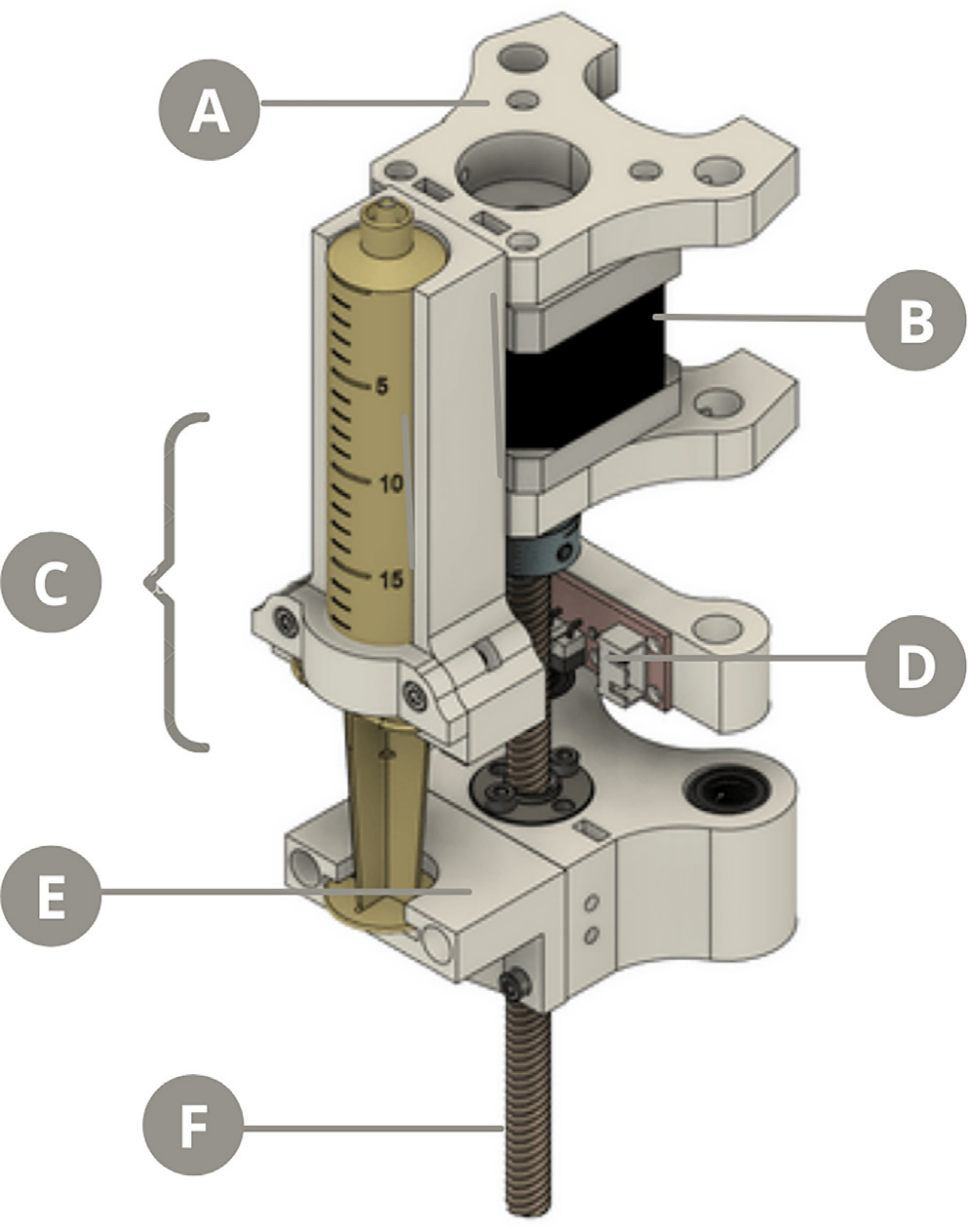
Exploded view diagram of the syringe holder. A) Step motor holder B) Nema 17 step motor C) Syringe holder D) End-stop switch E) Plunger holder F) Threaded rod.

The operating principle of the syringe pump is based on the vertical displacement of the plunger of a 20 mL syringe (CEGAMED®), which is fixed to a 3D printed case. The plunger is coupled to another plastic piece, whose movement is controlled by a Nema 17 step motor and limited by a mechanical end-stop switch (Fig. 3). The step motor is controlled using an A4988 driver through the firmware instruction *# define DEFAULT_AXIS_STEPS_PER_UNIT*. A value of 5960 steps in the vertical axis was defined to obtain a 3.5 mm linear movement that allows the syringe to eject 1±0.08 ml of liquid (see Firmware section).

**Fig. 4:**
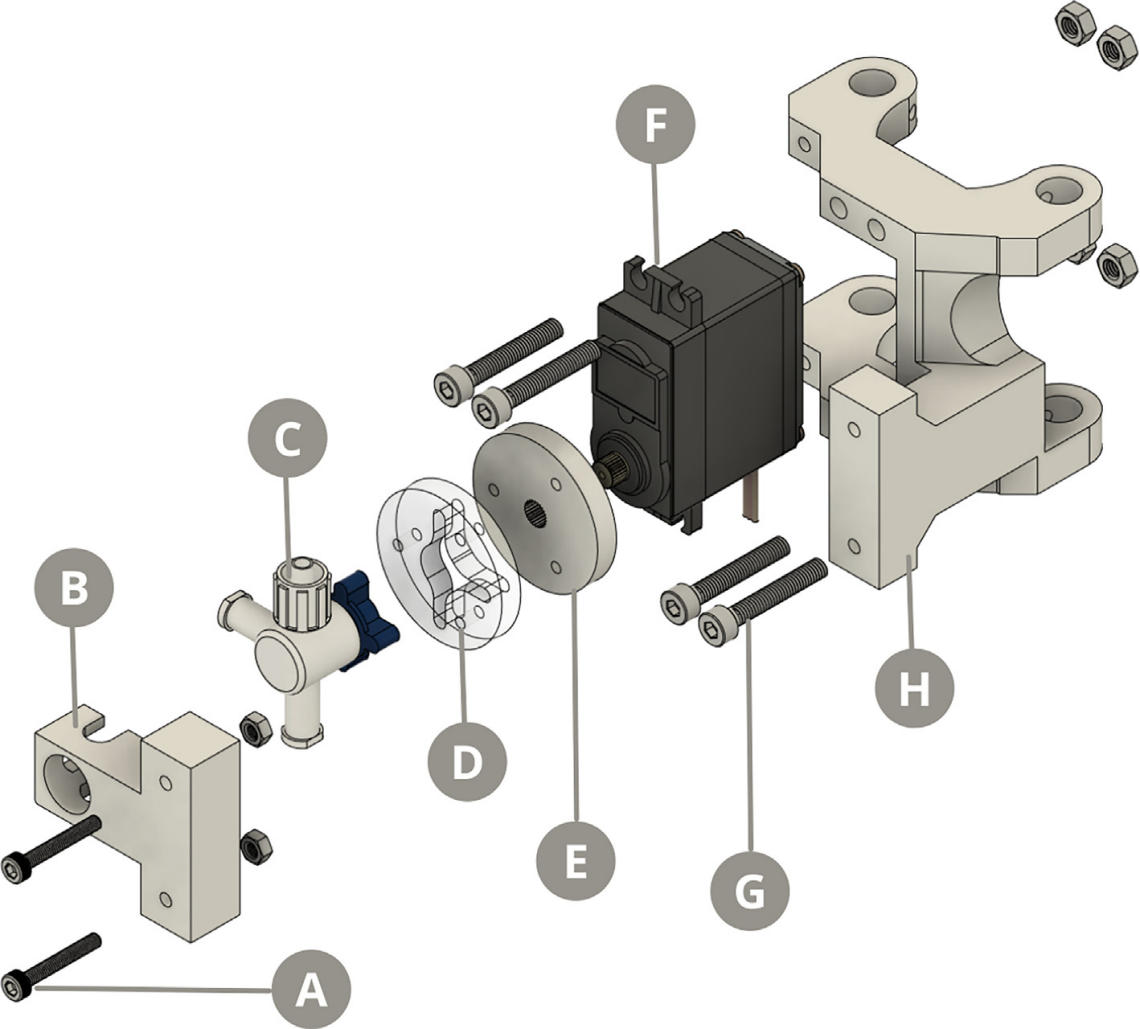
Exploded view diagram of the syringe pump. A) M3 screws B) Three-way stopcock fixer C) Three-way stopcock D) Valve adapter E) Servomotor adapter F) MG995 servomotor G) M4 screws H) Servomotor holder.

On the other hand, the flow valve comprises a three-way stopcock adapted to a MG955 servomotor that controls the inlet and outlet of fluids from both the containers in the reagent dispenser, and the reaction chamber (Fig. 4).

From top to bottom, the vacuum valve, reaction chamber, flow valve and syringe holder are assembled over a vertical structure made up of two metal rods (right structure in Fig. 1).

### Automated reagent dispenser

**Fig. 5:**
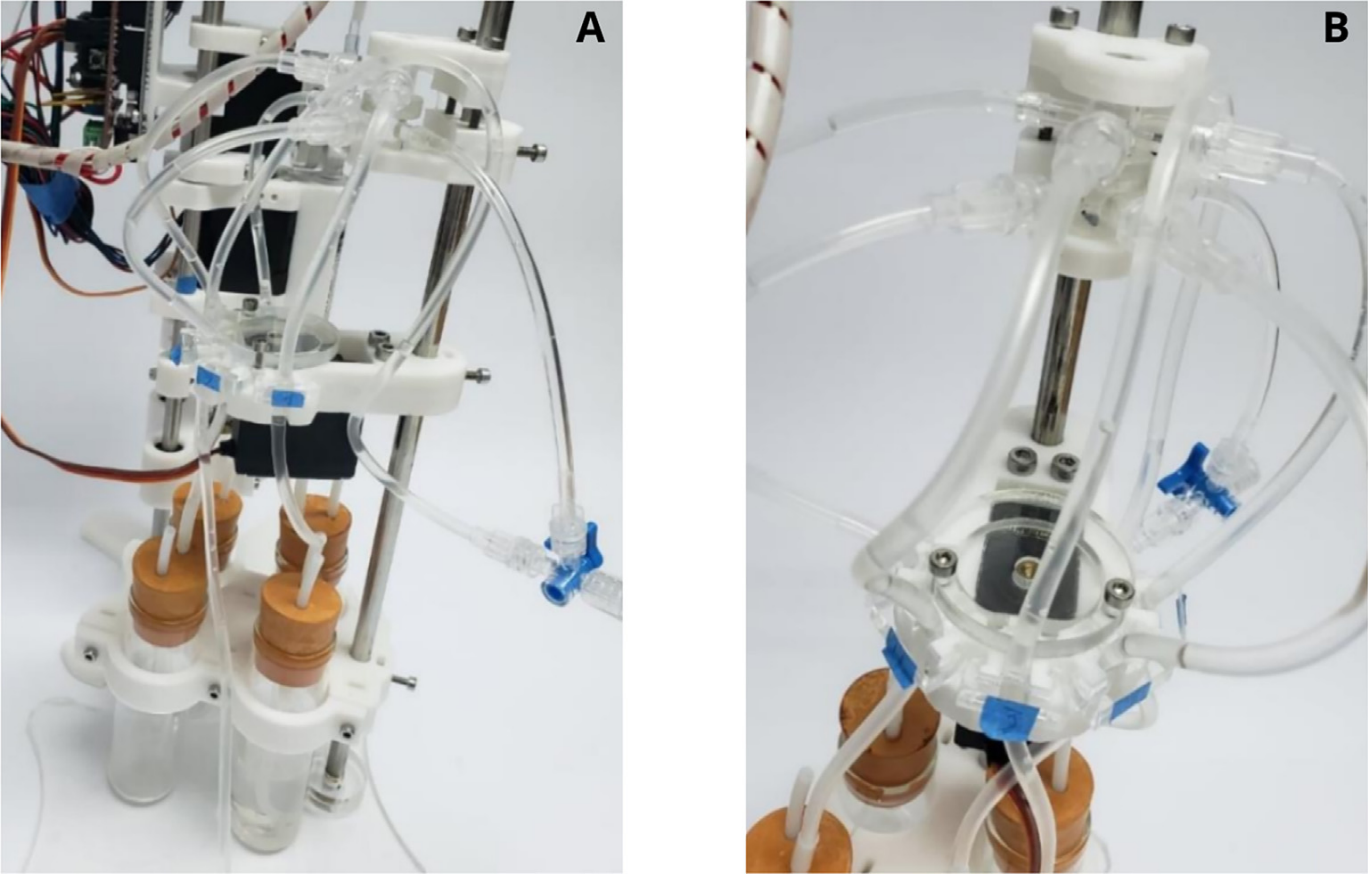
Overview of the reagent dispenser. A) Reagent containers B) Multiple access circuit (top) and five-way pinch valve (bottom).

**Fig. 6:**
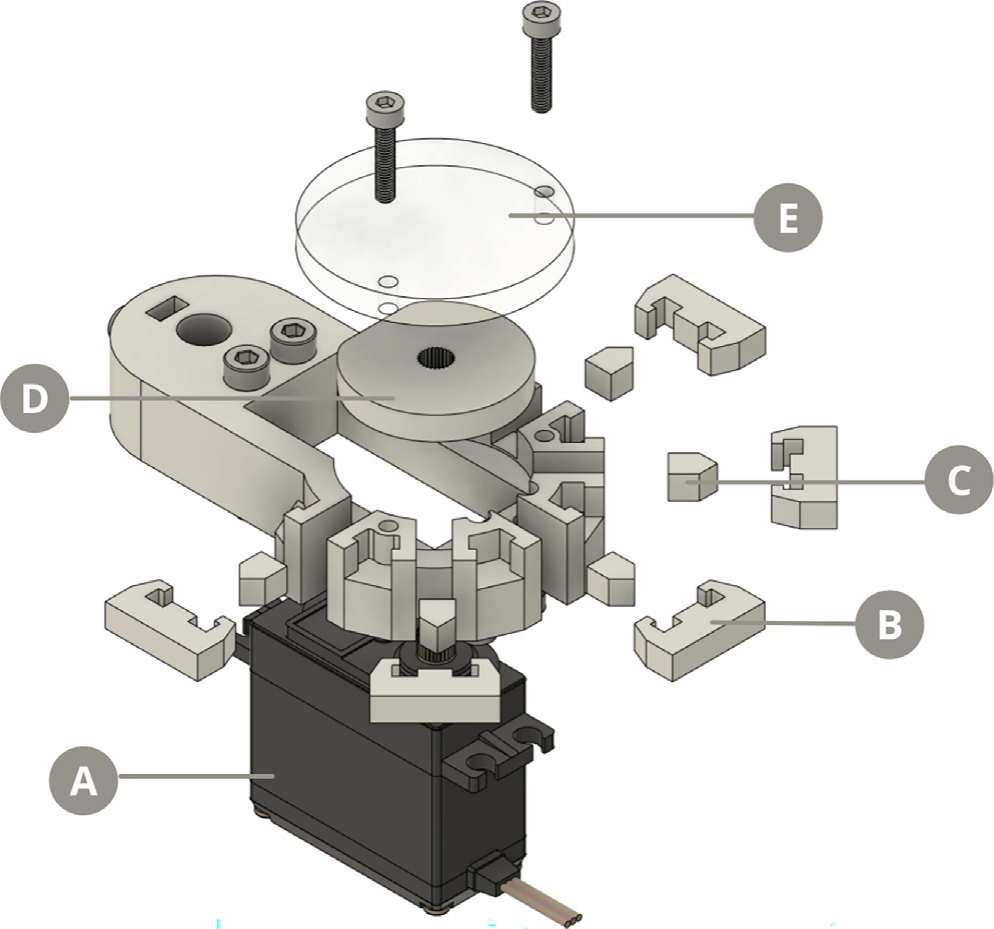
Exploded view diagram of the modified pinch valve. A) MG995 servomotor B) Valve clamp C) Block piston D) Block mechanism E) Top lid.

The reagent dispenser comprises three subcomponents fixed to a metal rod. From bottom to top, a 3D printed case was designed to hold four reagent containers: three corresponding to the DBM fabrication protocol (hydrochloric acid, hydrogen peroxide and isopropyl alcohol) and one for waste discharge (Fig. 5A). On top of this case, a modified version of the pinch valve from the open source turbidodast made from the instructions of the Klavin Lab (http://klavinslab.org/) was set to control the discharge of demineralization reagents (Fig. 6). A servomotor was integrated to the body of the pinch valve to control five valves: four connected to the containers and one connected to a high-flow access for washing solvents. Finally, a multiple access hose circuit was placed on top of the rod (Fig. 5B).

### Electronic circuit

**Fig. 7:**
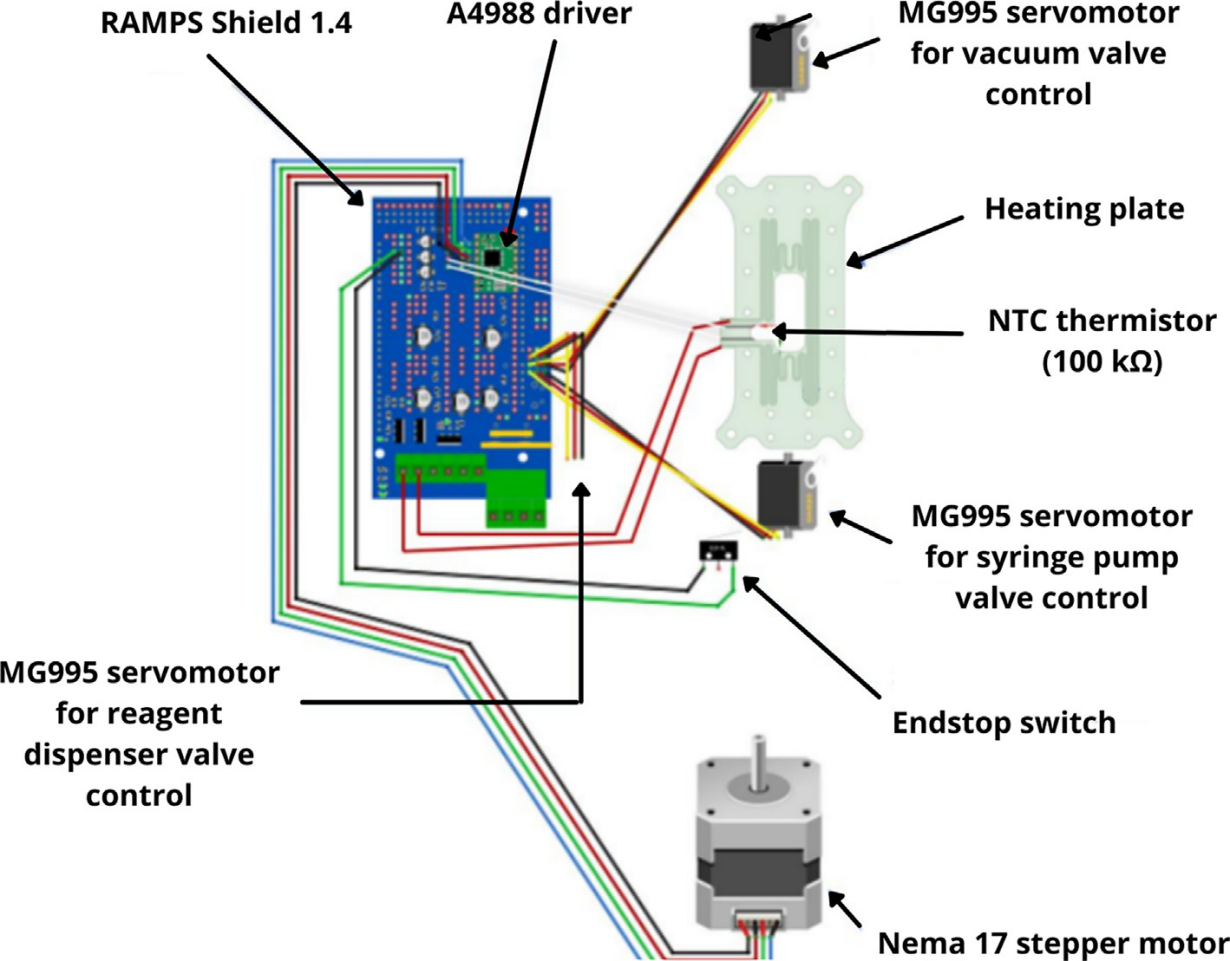
Circuit board layout, showing the connections between sensors and actuators with the RAMPS shield.

The electronic hardware was built upon the shield RAMP 1.4 due to its versatility and the incorporation of the MOSFET STB55NF06L that allows the temperature control of the reaction chamber (see Firmware section). The A4988 drivers are welded to the shield and allowed the control of the Nema 17 step motor and the three servomotors, as well as the NTC thermistor of the reaction chamber and the end-stop switch of the syringe pump (Fig. 7).

### Firmware

A modified version of the firmware Marlin 2.0.5.3 (https://marlinfw.org/) was used to control the perfusion system through G code commands described in Table 2.

**Table 2:** Commands used in the GUI and their translation to G code.

Control command	Description	G code
Emergency stop	Stop the perfusion systeḿs current operation	M112\r\n
**Syringe pump valve control**		
Zero Position	Set z-axis to its initial (zero) position	G28 Z0 F30\r\n
Loading	Enable connection between reagent dispenser and syringe	M280 P0 S0\r\n
Discharge	Enable connection between syringe and reaction chamber	M280 P0 S94\r\n
High flux	Enable connection between reagent dispenser and reaction chamber^a^	M280 P0 S180\r\n
**Syringe pump control**		
Send 10 mL	Positive vertical displacement to inject 0.1 mL of liquid	\rG91\r\nG1 Z-10 F30\r\nG90
Send 1 mL	Positive vertical displacement to inject 1 mL of liquid	\rG91\r\nG1 Z-1 F30\r\nG90
Send 0.1 mL	Positive vertical displacement to inject 10 mL of liquid	\rG91\r\nG1 Z-0.1 F30\r\nG90
Load 0.1 mL	Negative vertical displacement to load 0.1 mL of liquid	\rG91\r\nG1 Z0.1 F30\r\nG90
Load 1 mL	Negative vertical displacement to load 1 mL of liquid	\rG91\r\nG1 Z1 F30\r\nG90
Load 10 mL	Negative vertical displacement to load 10 mL of liquid	\rG91\r\nG1 Z10 F30\r\nG90
Send 10 mL slowly	Positive vertical displacement to inject 10 mL of liquid at a lower rate	\rG91\r\nG1 Z-10 F3\r\nG90
**Vacuum valve control**		
Open valve	Enable connection between air inlet and reaction chamber	M280 *P*1 S90\r\n
Vacuum connection	Enable connection between vacuum pump and reaction chamber	M280 *P*1 S170\r\n
**Multiple valve control**		
1 Waste discharge	Enable connection between waste discharge container and syringe pump valve	M280 *P*2 S180\r\n
2 Isopropyl alcohol	Enable connection between isopropyl alcohol container and syringe pump valve	M280 *P*2 S135\r\n
3 Saline solution	Enable connection between high-flux inlet and syringe pump valve	M280 *P*2 S90\r\n
4 Chloric acid	Enable connection between chloric acid container and syringe pump valve	M280 *P*2 S45\r\n
5 Hydrogen peroxide	Enable connection between hydrogen peroxide container and syringe pump valve	M280 *P*2 S0\r\n
**Temperature control**		
Heating 60 °C	Increase temperature of heating plate to 60 °C	M104 S60 T0\r\n
Heating OFF	Turn off the heating plate to allow cooling to room temperature	M104 S0 T0\r\n
Temperature reading	Display measurement from the NTC thermistor	M105\r\n

(^a^) Intended to be used for high-flux cleaning of the reaction chamber using PBS, loaded directly from the high-flux inlet in the reagent dispenser.

(^b^) To obtain the position of the syringés plunger with respect to the end-stop switch, we added a button named “Position”.

**Fig. 8:**
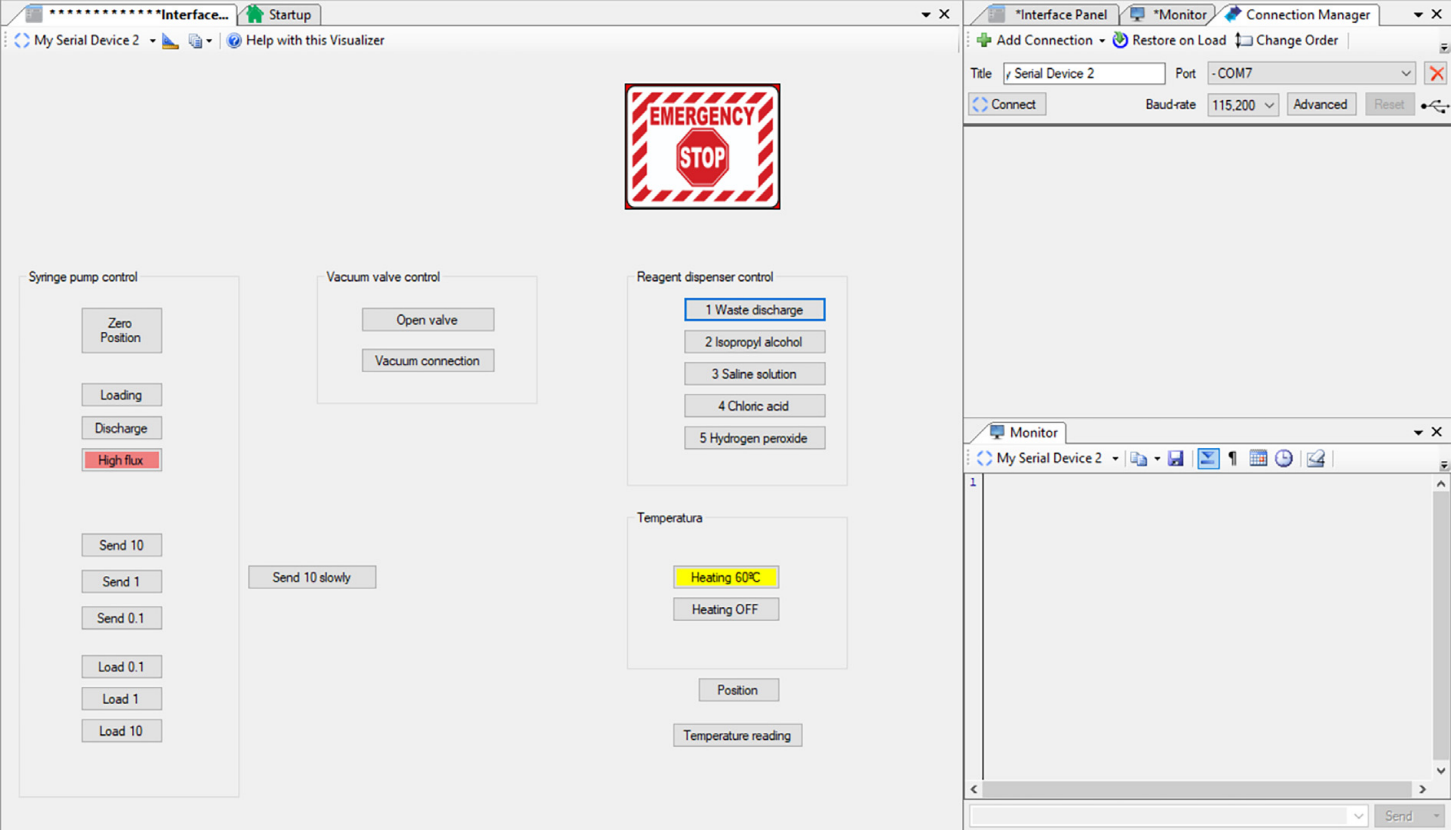
GUI of the perfusion system.

The GUI was developed using the software MegunoLink 1.32 (https://www.megunolink.com/) to control the developed system. The buttons are shown in Fig. 8, and its corresponding translation to G code is detailed in Table 2.

The temperature calibration of the heating plate was accomplished with the PID controller of an Arduino and the MOSFET STB55NF06L of the RAMPS for current regulation. The PID programming was achieved using the commands in the terminal of the Marlin firmware available in Supplementary Material 1 to limit the current of the heating plate.

## Design files summary

The 3D printing files were designed in Autodesk Fusion 360, while machined parts were designed in Ansys SpaceClaim. Both groups of files are available in STEP format for editing. **3D printed parts** were fabricated using PLA material, at 0.2 mm layer height and 60 % infill. **Machined parts** were fabricated with a CNC router or by laser cutting based on DXF files. The materials used for each piece are included in the description below Table 3. Both the firmware and the GUI, as well as the design files, can also be accessed through the Zenodo repository of the present project (https://doi.org/10.5281/zenodo.6795588).

**Table 3:** Design files of the perfusion system.

Design file name	File type	Open source license	Location of the file
**Flow reaction chamber**			
heating_plate	.stp file	BSD-2	Available with the article / https://doi.org/10.5281/zenodo.6795588
sil_cover	.stp file	BSD-2	Available with the article / https://doi.org/10.5281/zenodo.6795588
micro_reactor	.stp file	BSD-2	Available with the article / https://doi.org/10.5281/zenodo.6795588
main_lid	.stp file	BSD-2	Available with the article / https://doi.org/10.5281/zenodo.6795588
sil_joint	.stp file	BSD-2	Available with the article / https://doi.org/10.5281/zenodo.6795588
pla_lid	.stp file	BSD-2	Available with the article / https://doi.org/10.5281/zenodo.6795588
**Syringe pump & Vertical holder**			
top_lid	.stp file	BSD-2	Available with the article / https://doi.org/10.5281/zenodo.6795588
base	.stp file	BSD-2	Available with the article / https://doi.org/10.5281/zenodo.6795588
nema_holder	.stp file	BSD-2	Available with the article / https://doi.org/10.5281/zenodo.6795588
syringe_holder	.stp file	BSD-2	Available with the article / https://doi.org/10.5281/zenodo.6795588
syringe_fix	.stp file	BSD-2	Available with the article / https://doi.org/10.5281/zenodo.6795588
endstop_holder	.stp file	BSD-2	Available with the article / https://doi.org/10.5281/zenodo.6795588
plunger_stand	.stp file	BSD-2	Available with the article / https://doi.org/10.5281/zenodo.6795588
plunger_holder	.stp file	BSD-2	Available with the article / https://doi.org/10.5281/zenodo.6795588
servo_holder	.stp file	BSD-2	Available with the article / https://doi.org/10.5281/zenodo.6795588
servo_adapter	.stp file	BSD-2	Available with the article / https://doi.org/10.5281/zenodo.6795588
3valve_adapter	.stp file	BSD-2	Available with the article / https://doi.org/10.5281/zenodo.6795588
3valve_fix	.stp file	BSD-2	Available with the article / https://doi.org/10.5281/zenodo.6795588
**Automated reagent dispenser**			
container_body	.stp file	BSD-2	Available with the article / https://doi.org/10.5281/zenodo.6795588
container_fix	.stp file	BSD-2	Available with the article / https://doi.org/10.5281/zenodo.6795588
valve_body	.stp file	BSD-2	Available with the article / https://doi.org/10.5281/zenodo.6795588
valve_block	.stp file	BSD-2	Available with the article / https://doi.org/10.5281/zenodo.6795588
valve_cover	.stp file	BSD-2	Available with the article / https://doi.org/10.5281/zenodo.6795588
valve_clamp	.stp file	BSD-2	Available with the article / https://doi.org/10.5281/zenodo.6795588
piston_block	.stp file	BSD-2	Available with the article / https://doi.org/10.5281/zenodo.6795588
circuit_case	.stp file	BSD-2	Available with the article / https://doi.org/10.5281/zenodo.6795588
base	.stp file	BSD-2	Available with the article / https://doi.org/10.5281/zenodo.6795588

Flow reaction chamber: every piece is fabricated using a CNC router and laser cut, except *pla_lid*, which is 3D printed.

• ***heating_plate*** is a customized PCB circuit with the epoxy heating plate that is fixed to the back of the micro reactor with M3 screws. This piece can be either fabricated or bought from a local hardware store
• ***sil_cover*** is the silicone rubber cover located between the heating plate and the microreactor, or the microreactor and the main lid, both allowing the microscope slides to be accommodated.
• ***micro_reactor*** is the hollow customized PMMA block where the bone chips are placed for the demineralization process
• ***main_lid*** is the acrylic lid that is fixated to the anterior part of the microreactor with M3 screws
• ***sil_joint*** and ***pla_lid*** are the components that allow the Luer locks to fit in place

Syringe pump & Vertical holder: Every piece is 3D printed, except for the *3valve_adapter*, which is fabricated using laser cut.

• ***top_lid*** and ***base*** are the components that hold the two metal rods in place
• ***nema_holder*** is the component that fixates the Nema 17 step motor and aligns it with the threaded rod
• ***syringe_holder*** is the case where the 20 mL syringe is placed, which is adhered to the *nema_holder* piece
• ***syringe_fix*** is the component that fixates the syringe to the *syringe_holder* piece using M3 screws
• ***endstop_holder*** places the end-stop switch in the movement range of the *plunger_stand* piece
• ***plunger_stand*** is the piece whose vertical displacement is controlled by the step motor through the rotation of the threaded rod
• ***plunger_holder*** couples the *plunger_stand* movement with the syringés plunger
• ***servo_holder*** is the part that holds the servomotor to the metal rods of the vertical holder
• ***servo_adapter*** is the piece that connects the servomotor and the adapter of the three-way stopcock
• ***3valve_adapter*** is the acrylic piece that connects the three-way stopcock and the adapter of the servomotor
• ***3valve_fix*** is the part that fixates the three-way stopcock to the top of the syringe

Reagent dispenser: Every piece is 3D printed, except for the *base*, which is fabricated using laser cut.

• ***container_body*** is the part that holds the four reagent containers and fixes them to the metal rod
• ***valve_body*** is the part that holds the servomotor and through where the reagents hoses that come from the containers are fixated
• ***valve_block*** is the part that allows the inlet/outlet of one reagent hose by blocking the flow in the other hoses
• ***valve_cover*** is the acrylic part that joins the block part to the body of the valve
• ***valve_clamp*** is the part that holds the reagent hose to the body of the valve, while ***piston_block*** control the flow
• ***circuit_case*** holds the multiple-access circuit
• ***base*** holds the metal rod in place

## Bill of materials

To enable health personnel and researchers to build and use our perfusion system, we considered the need to purchase the minimum amount of external components. Most parts needed for assembling the system have been presented in the Design Files summary. Hence, the BOM mainly consists of screws, nuts and rods that should be available at any hardware store, as well as the electronic components. More specialized parts, such as the linear ball bearing, the shaft coupler, and the pillow block bearing, can be found online or in any store specialized in 3D printers. In fact, the RAMPS shield, drivers, stepper motors, metal rods, bearings and couplers can be obtained from a repurposed 3D printer.

The complete BOM can be found in Table 4.

**Table 4:** Bill of Materials (BOM).

Designator	Component	Number	Cost per unit ($)	Total cost ($)	Source of materials
Control board (Electronics) & General mechanics	Arduino Mega 2560 Rev3	1	37	37	Amazon
	RAMPS 1.4 Control Panel 3D Printer Control Board	1	8	8	Amazon
	A4988 Stepper Motor Driver	1	1.5	1.5	Alibaba
	Three-way stopcock	2	4	8	Amazon
Reaction chamber	M3 Stainless Steel Screws and Nuts	1	∼cents	15*	Local hardware store
	M4 Stainless Steel Screws and Nuts	1	∼cents	15*	Local hardware store
	100 K ohm NTC 3950 Thermistor (1 m / 34.9 in.)	1	2	2	Amazon
	Microscope slides	2	∼cents	10*	Local labware store
	0.2 μm syringe filter (ø = 25 mm)	1	13	13	Ebay
Syringe pump	20 mL syringe with Luer lock adapter	1	∼2	17*	Local labware store (CEGAMED)
	Endstop Mechanical Limit Switch	1	1.35	1.35	Alibaba
	Nema 17 Stepper motor	1	11	11	Amazon
	Linear Ball bearing (LM8UU)	2	3	6	Adafruit
	5 mm to 8 mm Flexible Shaft Coupling connector	1	10	10	Amazon
	Threaded Rod (ø = 8 mm, length: 450 mm)	1	12	12	Local hardware store
	Smooth rod (ø = 8 mm, length: 600 mm)	2	20	40	Local hardware store
	Brass T8 screw nut	1	10	10	Amazon
	KFL08 FL08 Self Alinear Pillow Block (ø = 8 mm)	1	5	5	Amazon
Reagent dispenser	Venoclysis equipment	2	2	4	Local labware store
	30 cm extension lines	5	1	5	Local labware store (Braun)
	Smooth rod (ø = 8 mm, length: 250 mm)	1	5	5	Local hardware store
	5-way connector for multiple injections	1	∼2	101*	Local labware/medical store (doctorshop)

(*) The total cost represents the commercial price of a box or kit containing the supplies needed for the fabrication of the system.

## Build instructions

The step-by-step procedure to build the perfusion system can be found in the official wiki of the project (https://sites.google.com/pucp.edu.pe/icoba-dbm-bioreactor/home). The additional tools required for the assembly include a set of screwdrivers, jumper cables, and soldering iron.

## Operation instructions

The *trabecular bone tissue pre-processing* section can be skipped if the researcher is not going to work with human tissue samples.

### Trabecular bone tissue pre-processing

The materials and supplies necessary to extract and recover trabecular bone tissue chips, according to the protocol developed by the Luis Vernaza Hospital [13], are listed in Table 5.

**Table 5:** Equipment and consumables used during the pre-processing protocol of trabecular bone chips fabrication.

Equipment	Model*
Oscillating saw	Stryker – GPL 650
Refrigerated centrifuge	Sigma – 3-16PK
Laminar flow biosafety cabinet class II type A2	Baker – SG603-HE-INT
Physiological buffered saline (PBS) solution	Gibco
Femoral condyles	Obtained from the Luis Vernaza Hospitaĺs Tissue Bank with informed consent of donors

(*) Any equipment with the same technical specifications will work for this step.

The validation and characterization of the present project were accomplished using trabecular bone chips obtained from femoral condyles of cadaveric female donors between 31 and 40 years and cadaveric male donors between 41 and 50 years (see Ethics statement) since they are expected to have optimal osteoinductive properties [23]. The following protocol for bone chip fabrication was implemented.

1. Defrost and thaw slowly the bone samples by transferring to a refrigerator between −4 to 8 °C for 12 h
2. In the biosafety cabinet, cut axially the bone samples with a thickness of 1 cm
3. Laminate bone as sheets of 15 × 20 × 5 mm (referred as chips) from the 1 cm-thick samples
4. Wash twice the chips as follows: Place in PBS and agitate for five minutes at 40 °C, then drain for three minutes
5. Centrifuge the chips at 1800 rpm
6. Keep at a designated quarantine freezer at −20 °C until further processing.

### DBM fabrication

The reagents necessary for the demineralization of the bone chips are detailed in Table 6.

**Table 6:** Consumables used during the DBM fabrication protocol.

Consumables	Brand
Chloric acid at 36 %*	J.T. Baker
Hydrogen peroxide at 30 %	Fisher Scientific
Isopropyl alcohol 100 %	Fisher Scientific
Saline solution 0.9 %	Baxter

(*) To prepare 1000 mL of hydrochloric acid solution at 0.5 N, we diluted 63.5 mL of chloric acid (J.T Baker®) in 300 mL of distilled water. Then, we add more distilled water up to 1000 mL.

#### Reagent dispenser loading

Fill the containers with the hydrochloric acid, hydrogen peroxide and isopropyl alcohol, respectively. Then, take another empty-one as waste discharge and place the four containers in their assigned holders. Place the tubing through the cap of each container. Clockwise, the order of the containers should be (1) waste container, (2) isopropyl alcohol, (3) high-flux inlet for washing solutions, (4) chloric acid, and (5) hydrogen peroxide (see Table 2. Multiple valve control).

#### Bone chip loading

Transfer the bone chip from the quarantine freezer into a fridge for 12 h for thawing. Once ready, take one bone chip and place it inside the empty chamber in the microreactor using sterile tweezers.

#### Controlling the perfusion system

Follow the steps detailed in Table 7. For Step 3, saline solution should be loaded from the high-flow access hose.

**Table 7:** Step-by-step operation instructions.

Step	Description	Observation
1	Connect the system to the power supply. Next, in the GUI, press the command *Zero Position*. Then, press the command *Open valve*. After (?) min, press the command *Vacuum connection*	Set the system to its default configuration and execute the pressurization step
2	Press the command *Heating 60 °C*	Initial washing configuration step
3	Press the command *High flux* and *Saline solution*	
4	Press the command *Send 10 mL slowly*	This step should be repeated **three times**
5	Press the command *Waste discharge*	Send waste liquid into its respective container
6	**Valve configuration:** Press the command *Loading* and the command *Hydrogen peroxide*. Then, press the command *Discharge* **Hydrogen peroxide:** Then, press the command *Load 10 mL*. Finally, press the command *Send 10 mL*	This step should be repeated **twice.** Skip the command ***valve configuration*** in the second iteration
7.	Repeat Step 3–5	Washing step
8	**Valve configuration:** Press the command *Loading* and the command *Isopropyl alcohol*. Then, press the command *Discharge* **Isopropyl alcohol:** Then, press the command *Load 10 mL*. Finally, press the command *Send 10 mL*	This step should be repeated **twice.** Skip the command ***valve configuration*** in the second iteration
9	Repeat Step 3–5	Washing step
10	**Valve configuration:** Press the command *Loading* and the command *Chloric acid*. Then, press the command *Discharge* **Chloric acid:** Then, press the command *Load 10 mL*. Finally, press the command *Send 10 mL*	This step should be repeated **twice.** Skip the command ***valve configuration*** in the second iteration
10	Repeat Step 3	Final washing step
11	Press the command *Send 10 mL slowly*	This step should be repeated **five times**
12	Press the command *Heating OFF*. When the reaction chamber cools down, press the command *Open valve*	Configuration to withdraw the DBM chip graft from the reaction chamber

## Validation and characterization

### Methodology

#### Quantification of residual lipids and calcium

To quantify residual lipids, eleven experimental units were generated at varying concentrations of hydrogen peroxide and isopropyl alcohol (see Supplementary Material 2). Likewise, for the residual calcium assay, eleven experimental units were processed at varying times and temperatures (see Table 8). The order of experimentation was chosen randomly, under the same environmental conditions (25 °C and relative humidity of 50 ± 10 %). The vacuum for all experimental units was set at – 700 mmHg. The reagents belonged to the same lot, and the experiments were performed the same day to minimize environmental and operative variability.

**Table 8:** Combination of factors to evaluate residual lipids and calcium.

**Residual lipid assay**			
**Experimental unit**	**Randomized order**	**Factor 1**	**Factor 2**
		**A:** Hydrogen peroxide (%)	**B:** isopropyl alcohol (IPA, %)
1	7	10	50
2	1	25	50
3	11	10	90
4	10	25	90
5	4	7	70
6	6	30	70
7	3	17	42
8	9	17	100
9	8	17	70
10	5	17	70
11	2	17	70
**Residual calcium assay**			
**Experimental unit**	**Randomized order**	**Factor 1**	**Factor 2**
		**A:** Temperature (°C)	**B:** Time (minutes)
1	9	25	30
2	1	60	30
3	2	25	120
4	6	60	120
5	11	17	75
6	10	67	75
7	3	42	11
8	5	42	140
9	4	42	75
10	7	42	75
11	8	42	75

At the end of each experiment, the DBM samples were placed to dry in an oven at 120 °C for 8 h. Then, the samples were weighted in an analytical scale to measure their dry weight (mg). Next, the samples were placed in a test tube and hydrolyzed with 1 mL of HCl at 6 N in a water bath at 40 °C until the tissue was hydrolyzed. Once dissolved, the sample was diluted in a beaker with 99 mL of distilled water.

**Quantification of residual calcium.** For this assay, we used electrochemiluminescence to measure the residual calcium in the DBM by using the kit CALCIUM Gen.2 in the Roche Cobas C501 Chemistry Analyzer. The analytical protocol was based on manufacturers recommendations for urine samples to obtain results expressed as total calcium (mg/dL). In addition, if the sample yields high values of calcium (e.g normal human urine samples), a 1:5 dilution was used to stay within the detection range.

The results will be calculated using the following equation:

$$\%Ca^{+2}=\frac{Ca_{\mathrm{Total}}}{W_{\mathrm{tissue}}}\times 100$$

where $Ca_{\mathrm{Total}}$ = total calcium, expressed in mg/dL, and $W_{\mathrm{tissue}}$ = dry weight of the tissue sample, expressed in mg/dL.

**Quantification of residual lipids.** For this assay, we used a manual technique, following the total lipid quantification protocol from the Luis Vernaza Hospital using the Spinreac Total Lipid Colorimetric kit. Following the protocol detailed by the manufacturer, the sample is analyzed by spectrophotometry (520 nm) and compared to a standard curve.

The results will be calculated using the following equation:

$$\%lipid=\frac{L_{\mathrm{Total}}}{W_{\mathrm{tissue}}}\times 100$$

where $L_{\mathrm{Total}}$ = total lipids, expressed in mg/dL, and $W_{\mathrm{tissue}}$ = dry weight of the tissue sample, expressed in mg/dL.

#### Optimization using response surface methodology (RSM) of design of experiments (DoE)

Following the rationale for the design and fabrication of the perfusion system, the following parameters should be optimized:

• For cost-effectiveness, the amount of hydrogen peroxide and isopropyl alcohol needed to obtain a lipid concentration < 5 % in the DBM [24] was optimized
• To avoid the denaturalization of relevant proteins in the DBM, the time and exposure to the acidic treatment was optimized.

In this context, the RSM was applied for two experiments: (1) the characterization of the relationship between the final concentration of residual lipids, and the concentration of hydrogen peroxide and isopropyl alcohol, and (2) the characterization of the relationship between demineralization time and temperature in the reaction chamber to obtain a minimum of 8 % of residual calcium [25].

### Optimization of the protocol based on lipid and calcium removal

#### Optimization of the lipid removal process

The results from the residual lipid assay of the eleven experimental units are shown in Table 9. We found a range of total lipids in the DBM samples with a minimum of 2.3 % and a maximum of 18.4 %. Furthermore, the quadratic model is found to be statistically significant (p < 0.0001). Accordingly, the model shows that both the hydrogen peroxide and the isopropyl alcohol have a significant impact in the total lipid percentage. In fact, the isopropyl alcohol yielded a higher effect in the total lipid calculation than the peroxide hydrogen (see Supplementary Material 3).

**Table 9:** Results from the total lipid assays.

**Experimental unit (by order)**	**Factor 1**	**Factor 2**	**Total lipid (%)**
	**A:** Hydrogen peroxide (%)	**B:** IPA (%)	
2	25	50	13
11	17	70	5.4
7	17	42	18.4
5	7	70	6.9
10	17	70	5.9
6	30	70	3.6
1	10	50	15
9	17	70	6.3
8	17	100	2.6
4	25	90	2.3
3	10	80	2.4

For cost-effectiveness, we performed an RSM optimization to find the adequate concentration of hydrogen peroxide and isopropyl alcohol that yields a total lipid percentage of < 5 % and uses less reagents. The results from the calculation yielded a combination of 12 % of peroxide hydrogen and 80 % of isopropyl alcohol (see Supplementary Material 3). Accordingly, three experimental units were fabricated and tested under the optimized parameters, obtaining a total lipid percentage of 4 % (see Table 10).

**Table 10:** Total lipid percentage with the optimized parameters (12 % hydrogen peroxide and 80 % isopropyl alcohol) calculated with RSM, for n = 3.

	**Total lipids (%)**
**Experimental unit 1**	4.1
**Experimental unit 2**	3.8
**Experimental unit 3**	4.3
**Calculated mean**	4.17959
**Obtained mean**	4.066667

#### Optimization of the temperature and time of demineralization

The results from the residual calcium assay of the eleven experimental units are shown in Table 11. We found a range of residual calcium in the DBM samples with a minimum of 2 % and a maximum of 48 %. Moreover, the quadratic model is found to be highly significant (p < 0.09). This model shows that the acidic treatment time is more significant than the reaction temperature, and that both have an apparent inverse correlation (i.e) the increase in temperature correlates with less demineralization time.

**Table 11:** Results from the total calcium assay.

**Experimental unit (by order)**	**Factor 1**	**Factor 2**	**Total calcium (%)**
	**A:** Temperature (°C)	**B:** Time (minutes)	
2	60	30	22
3	25	120	16
7	42	11	48
9	42	75	16
8	42	140	5
4	60	120	2
10	42	75	18
11	42	75	17
1	25	30	42
6	67	75	2
5	17	75	29

For further analysis, we performed an RSM optimization to estimate an optimized demineralization time with a minimized temperature value to avoid the degradation of the DBM product. The result of the calculation yielded 120 min of reaction at a constant temperature of 45 °C (see Supplementary Material 4). Accordingly, three experimental units were fabricated and tested under the optimized parameters, obtaining a final calcium percentage of 7 % (see Table 12).

**Table 12:** Total calcium percentage with the optimized parameters (Temperature: 45 °C, Time: 120 min) calculated with RSM, for n = 3.

	**Residual calcium (%)**
**Experimental unit 1**	7.2
**Experimental unit 2**	6.8
**Experimental unit 3**	6.9
**Calculated mean**	6.99
**Obtained mean**	6.96

### Confirmation of decellularization and collagen structural integrity of chips

**Fig. 9:**
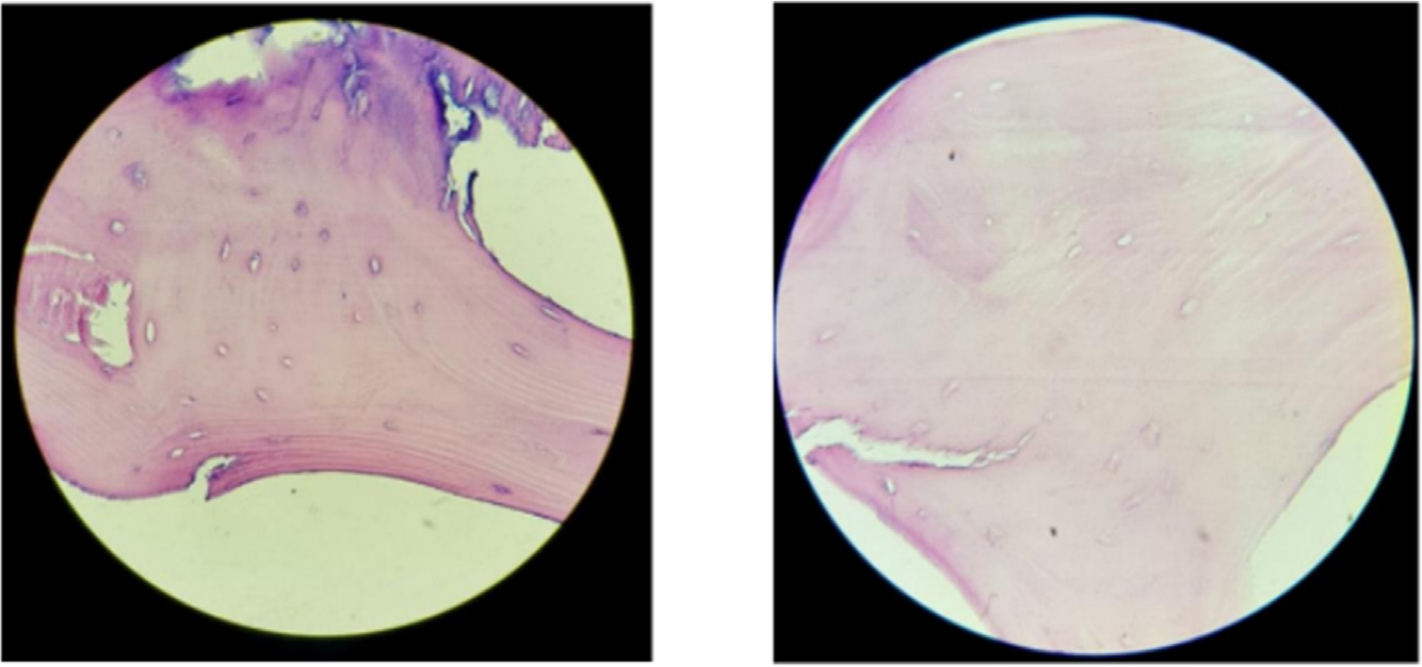
Histological analysis of a DBM sample. We can observe bone trabeculae without cellular components and an intact collagen matrix.

To validate the manufacturing protocol, the DBM samples were observed in an inverted microscope to determine the presence of cellular debris and the structural integrity of the collagen matrix. First, the DBM samples were dehydrated and embedded in a paraffin block to obtain samples of 5-10μm thickness. Then, samples were stained with Hematoxylin and Eosin, and examined in a bright-light inverted microscope (Fig. 9).

### Temperature calibration

**Fig. 10:**
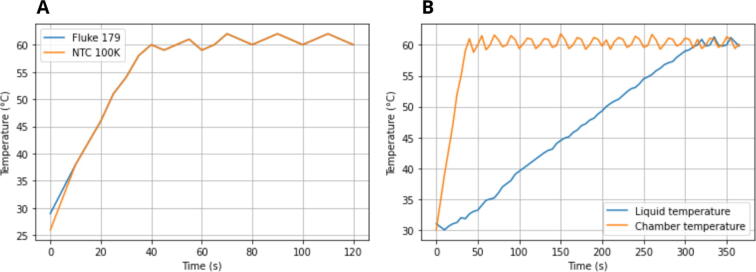
Temperature calibration. A) Comparison between the response of the 100 K NTC thermistor and the thermocouple of the Fluke 179 Digital Multimeter B) Total time for the reaction chamber to increase the temperature of saline solution, from room temperature to 60 °C.

**Heating plate.** To validate the temperature regulation in the heating plate, we used the thermocouple embedded in the Fluke 179 Digital Multimeter to generate a reference measurement of the temperature of the heating plate. Then, we compared this reference with the measurements obtained by the NTC thermistor located in the base of the heating plate. The response of the thermistor with respect to the reference measurement presented a difference of ± 0.4 °C (Fig. 10A).

**Reaction chamber.** To characterize the capacity of the flow reaction chamber to increase the temperature of the reagents, we remove the front cover of the reaction chamber and immerse the thermocouple into the reaction chamber. Then, we load saline solution into the flow reaction chamber, as detailed in the *Operation instructions* section, measuring the time for the liquid to match the temperature of the reaction chamber, given by the NTC thermistor. This assay was repeated 5 times to calculate a time average. In this way, we obtained a mean of 6 min for the reaction chamber to increase the temperature of the liquid from 31 °C to 60 °C (Fig. 10B).

## Conclusion

The ultimate goal of the present project was to create a cost-effective and highly reproducible perfusion system for the medium-scale production of demineralized bone matrix for tissue engineering applications. The overall setup includes a unidirectional flow reaction chamber, a modular syringe pump and an automated reagent dispenser, which enable the ideal experimental conditions, in terms of temperature, pressure and reagent handling, for trabecular bone demineralization recovered from human cadaveric femoral condyles. The system was successfully validated by executing electrochemiluminescence and colorimetric protocols, as well as histological analysis and temperature calibrations. The first three experiments validated the devicés ability to support the proposed demineralization protocol, which yielded values of residual lipids and calcium phosphates within the standardized ranges, and maintained the structural integrity of the resulting scaffold. Similarly, the last experiment demonstrated the capacity of the system to maintain a stable value of temperature necessary for the demineralization reaction to take place. Furthermore, to optimize the parameters involved in the DBM fabrication process, the Response Surface Methodology was applied, resulting in the following combination: 5 pulses per minute of saline solution for the washing stage, a combination of 12 % of peroxide hydrogen and 80 % of isopropyl alcohol for the lipid and debris removal stage, and chloric acid at 0.5 N for the demineralization stage, at a constant temperature ranging from 45 °C to 60 °C for 120 min.

As designed, the perfusion system is able to produce one DBM chip every 120 min, which is enough for an initial validation of the efficiency of the demineralization protocol and the performance of the system itself. However, to meet the increasing need for DBM grafts for large bone reconstructive surgery at the Luis Vernaza Hospital, we have designed (but not tested) a microreactor that contains an array of 16 parallel chambers (available in Github). Moreover, the present project used bone tissue samples only from femoral condyles. Further research is needed to explore the application of the perfusion system for the demineralization of bone tissue samples from other locations of the human body that are commonly used for bone grafting, such as the iliac crest and the vertebrae. Finally, we encourage the study of the correlation between the physicochemical parameters controlled by the developed system (i.e reaction time, temperature, vacuum, and reagents concentration and flow) and the osteoinductive capacity of the DBM chips.

## Ethics statements

To validate the present hardware, we have used trabecular bone tissue from cadaveric human subjects that were acquired following a clinical protocol approved by the Ethics Board of the Luis Vernaza Hospital (HLV-DIM-DFO-0001).

## Funding

This research did not receive any specific grant from funding agencies in the public, commercial, or not-for-profit sectors.

## CRediT authorship contribution statement

**Winston Jaramillo-Cañas:** Methodology, Software, Validation, Formal analysis. **Frank Britto-Bisso:** Methodology, Writing – original draft, Visualization. **Cesar Fernandez-Valiente:** Conceptualization. **Fanny L. Casado:** Conceptualization, Writing – review & editing, Supervision.

## Declaration of Competing Interest

The authors declare that they have no known competing financial interests or personal relationships that could have appeared to influence the work reported in this paper.
