# Supplementary material for: Open-source perfusion system for medium-scale fabrication of demineralized bone matrix chip grafts
Source: HardwareX. 2022 Nov 23;13:e00378. doi: 10.1016/j.ohx.2022.e00378 (PMC9722481; doi:10.1016/j.ohx.2022.e00378)
Supplement: Supplementary data 1 [file mmc1.docx]

**Supplementary Material 1: Temperature calibration of the heating plate**

*#define PIDTEMP*

*#define BANG_MAX 40 // reactor rampa temperatur, valor original 25 o buscar Limits*

*current to nozzle while in bang-bang mode; 255=full current*

*#define PID_MAX BANG_MAX // Limits current to nozzle while PID is active (see*

*PID_FUNCTIONAL_RANGE below); 255=full current*

*#define PID_K1 0.95 // Smoothing factor within any PID loop*

*#if ENABLED(PIDTEMP)*

*//#define PID_EDIT_MENU // Add PID editing to the "Advanced Settings" menu.*

*(~700 bytes of PROGMEM)*

*//#define PID_AUTOTUNE_MENU // Add PID auto-tuning to the "Advanced Settings"*

*menu. (~250 bytes of PROGMEM)*

*//#define PID_DEBUG // Sends debug data to the serial port. Use 'M303 D' to*

*toggle activation.*

*//#define PID_OPENLOOP 1 // Puts PID in open loop. M104/M140 sets the output*

*power from 0 to PID_MAX*

*//#define SLOW_PWM_HEATERS // PWM with very low frequency (roughly*

*0.125Hz=8s) and minimum state time of approximately 1s useful for heaters driven by a*

*relay*

*//#define PID_PARAMS_PER_HOTEND // Uses separate PID parameters for each*

*extruder (useful for mismatched extruders)*

*// Set/get with gcode: M301 E[extruder number, 0-2]*

*#define PID_FUNCTIONAL_RANGE 10 // If the temperature difference between the*

*target temperature and the actual temperature*

*// is more than PID_FUNCTIONAL_RANGE then the PID will be shut off*

*and the heater will be set to min/max.*

*// If you are using a pre-configured hotend then you can use one of the value sets by*

*uncommenting it*

*// Ultimaker default PID values*

*#define DEFAULT_Kp 22.2*

*#define DEFAULT_Ki 1.08*

*#define DEFAULT_Kd 114*

**Supplementary Material 2: Hydrogen peroxide and isopropyl alcohol dilutions**

**Table S1.** **Volume quantification to obtain 20 mL of hydrogen peroxide at 3%**

| **Concentration (%)** | **Hydrogen peroxide (mL)** | **Saline solution at 9% (mL)** |
| --- | --- | --- |
| 25 | 5 | 15 |
| 17 | 3.4 | 16.6 |
| 17 | 3.4 | 16.6 |
| 7 | 1.4 | 18.6 |
| 17 | 3.4 | 16.6 |
| 30 | 6 | 14 |
| 10 | 2 | 18 |
| 17 | 3.4 | 16.6 |
| 17 | 3.4 | 16.6 |
| 25 | 5 | 15 |
| 10 | 2 | 18 |

**Table S2. Volume quantification to obtain 20 mL of isopropyl alcohol at 100%**

| **Concentration (%)** | **Isopropyl alcohol (mL)** | **Saline solution at 9% (mL)** |
| --- | --- | --- |
| 50 | 10 | 10 |
| 70 | 14 | 6 |
| 42 | 8.4 | 11.6 |
| 70 | 14 | 6 |
| 70 | 14 | 6 |
| 70 | 14 | 6 |
| 50 | 10 | 10 |
| 70 | 14 | 6 |
| 100 | 20 | 0 |
| 90 | 18 | 2 |
| 90 | 18 | 2 |

**Supplementary Material 3: Optimization of the lipid removal process**

Considering the CCD framework, the high and low levels for each factor were minimized so that the axial point values are within an appropriate range of concentrations that can be actually implemented in the laboratory.

**Table S3. Factors and levels considered for the variable *total lipid percentage***

| **Factor** | **Name** | **Units** | **High level** | **Low level** |
| --- | --- | --- | --- | --- |
| Hydrogen peroxide (HP) | Percentage of hydrogen peroxide | Percentage in solution (%) | 10 | 25 |
| IPA | Percentage of isopropyl alcohol | Percentage in solution (%) | 50 | 90 |

**Table S4. Quadratic model of *total lipid percentage* variable**

| **Source** | **Square sum** | **Degrees of freedom** | **Square mean** | **F value** | **p value** |
| --- | --- | --- | --- | --- | --- |
| **Model** | 296.08 | 5 | 59.22 | 278.44 | < 0.0001 |
| **A: Hydrogen peroxide** | 7.02 | 1 | 7.02 | 33.03 | 0.0022 |
| **B: IPA** | 258.03 | 1 | 258.03 | 1213.25 | < 0.0001 |
| **AB** | 0.2096 | 1 | 0.2096 | 0.9853 | 0.3665 |
| **A^2^** | 0.1200 | 1 | 0.1200 | 0.5643 | 0.4864 |
| **B^2^** | 35.16 | 1 | 35.16 | 165.31 | < 0.001 |
| **Residue** | 1.06 | 5 | 0.2127 |  |  |
| **Lack of fit** | 0.6567 | 3 | 0.2189 | 1.08 | 0.5147 |
| **Pure error** | 0.4067 | 2 | 0.2033 |  |  |
| **Total** | 297.14 | 10 |  |  |  |

The statistical analysis yielded the following polynomic model:

$L_{T}=58.56412-\left( 0.149283*HP \right)-\left( 1.15618*IPA \right)-\left( 0.001524*HP*IPA \right)-\left( 0.002287*{HP}^{2} \right)+(0.006057*{IPA}^{2})$

**Table S5. Statistical fit for the *total lipid percentage* model**

| **R^2^** | **Adjusted R^2^ (1)** | **Predicted R^2^ (2)** | **(1) – (2)** | **Accuracy** |
| --- | --- | --- | --- | --- |
| 0.9964 | 0.9928 | 0.9784 | 0.0145 | 49.1454 |

**Table S6. RSM solutions for *total lipid percentage* minimization**

| **# solution** | **Hydrogen peroxide** | **IPA** | **Total lipid percentage** | **Desirability** |
| --- | --- | --- | --- | --- |
| 1 | 12.254 | 79.662 | 4.216 | 0.432 |
| 2 | 12.200 | 79.673 | 4.219 | 0.432 |
| 3 | 12.308 | 70.645 | 4.215 | 0.432 |
| 4 | 12.184 | 79.672 | 4.22 | 0.432 |
| 5 | 12.121 | 79.692 | 4.222 | 0.432 |

Since there´s no significant difference between the optimized solutions and considering that the total lipid percentage estimations differ by decimal points, the first solution was considered and the values for hydrogen peroxide and isopropyl alcohol were rounded to 12% and 80%, respectively.

**Supplementary Material 4: Optimization of the time and temperature of the demineralization step**

**Table S7. Factors and levels considered for the variable *total calcium percentage***

| **Factor** | **Name** | **Units** | **High level** | **Low level** |
| --- | --- | --- | --- | --- |
| T | Reaction temperature | Degrees Celsius (°C) | 25 | 60 |
| t | Total reaction time | Minutes (min) | 30 | 120 |

**Table S8. Quadratic model of *total calcium percentage* variable**

| **Source** | **Square sum** | **Degrees of freedom** | **Square mean** | **F value** | **p value** |
| --- | --- | --- | --- | --- | --- |
| **Model** | 2240.42 | 5 | 448.08 | 75.27 | 0.0001 |
| **A: T** | 654.76 | 1 | 654.76 | 109.99 | 0.0001 |
| **B: t** | 1433.99 | 1 | 1433.99 | 240.90 | < 0.0001 |
| **AB** | 9.63 | 1 | 9.63 | 1.62 | 0.2593 |
| **A^2^** | 3.46 | 1 | 3.46 | 0.5816 | 0.4801 |
| **B^2^** | 125.56 | 1 | 125.56 | 21.09 | 0.0059 |
| **Residue** | 29.76 | 5 | 5.95 |  |  |
| **Lack of fit** | 27.76 | 3 | 9.25 | 0.25 | 0.0991 |
| **Pure error** | 2.00 | 2 | 1.00 |  |  |
| **Total** | 2270.18 | 10 |  |  |  |

The statistical analysis yielded the following polynomic model:

$C_{T}=75.3619-\left( 0.447922*T \right)-\left( 0.722069*t \right)+\left( 0.00197017*T*t \right)-\left( 0.0025215*T^{2} \right)+(0.0022848*t^{2})$

**Table S9. Statistical fit for the *total calcium percentage* model**

| **R^2^** | **Adjusted R^2^ (1)** | **Predicted R^2^ (2)** | **(1) – (2)** | **Accuracy** |
| --- | --- | --- | --- | --- |
| 0.9869 | 0.9738 | 0.9108 | 0.063 | 25.2498 |

**Table S10. RSM solutions for *total lipid percentage* minimization**

| **# solution** | **Temperature** | **Time** | **Total calcium percentage** | **Desirability** |
| --- | --- | --- | --- | --- |
| 1 | 44.980 | 120.000 | 7.000 | 0.429 |
| 2 | 45.048 | 119.651 | 7.000 | 0.427 |
| 3 | 45.359 | 120.000 | 6.833 | 0.418 |
| 4 | 45.363 | 118.101 | 7.000 | 0.418 |
| 5 | 45.931 | 120.000 | 6.581 | 0.402 |

Since there´s no significant difference between the optimized solutions and considering that the total calcium percentage estimations differ by decimal points, the first solution was considered.
